# Supplementary material for: Mechano-modulatory synthetic niches for liver organoid derivation
Source: Nat Commun. 2020 Jul 10;11:3416. doi: 10.1038/s41467-020-17161-0 (PMC7351772; doi:10.1038/s41467-020-17161-0)
Supplement: Supplementary file 1 — Supplementary Information [file 41467_2020_17161_MOESM1_ESM.pdf]

Supplementary Information

Sorrentino et al.

Mechano-modulatory synthetic niches for liver organoid derivation

Supplementary Figure 1:

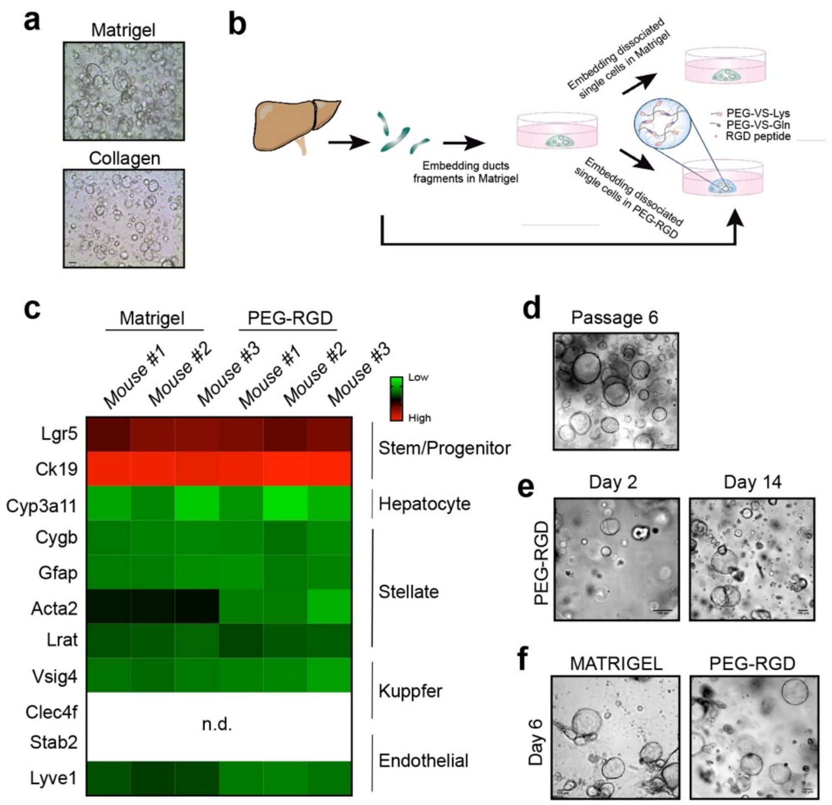

Supplementary Figure 1. Liver organoid growth in Matrigel and PEG | (a) Representative picture of mouse liver progenitor cells 6 days after embedding in 10µL droplet of Matrigel or Type I Collagen. Scalebar : 100 µm. **(b)** Schematic of the protocol for generating mouse liver organoid cell lines in Matrigel or, directly, in PEG-RGD hydrogels. **(c)** mRNA levels of markers of different cell types were analysed by qRT-PCR in liver organoids maintained in expansion medium. The heatmap represents ΔCt values as described in the method section. **(d)** Representative picture of liver organoids embedded in PEG-RGD at the indicated passage. **(e)** Representative picture of liver organoids embedded in PEG-RGD for the indicated times. **(f)** PEG-RGD hydrogel provides a stable matrix for long term culture of hepatic organoids, while Matrigel already softened after 6 days of culture. Scale bars: 100 µm. Micrographs are representative of three independent experiments. Source data are provided as a Source Data file.

Supplementary Figure 2:

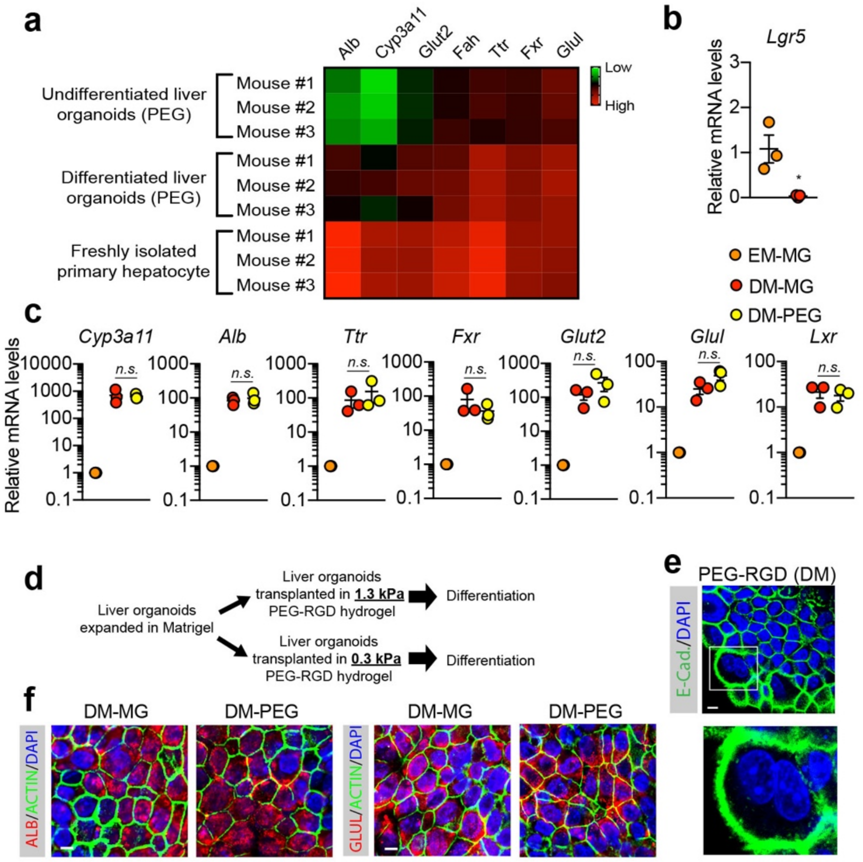

Supplementary Figure 2. Differentiation of liver organoids into hepatocyte-like cells | (a) mRNA levels of markers of hepatocyte were analysed by qRT-PCR in freshly isolated hepatocytes or in liver organoids embedded in PEG-RGD (PEG) and maintained in expansion (undifferentiated organoids) or differentiation (differentiated organoids) medium. The heatmap represents ΔCt values as described in the method section. **(b)** Lgr5 mRNA levels were analysed by qRT-PCR in liver organoids maintained in differentiation medium (DM). Graph show individual data points derived from n=3 independent experiments and means ± SEM. (P=0.0273). **(c)** Gene expression was analysed by qRT-PCR in liver organoids derived in Matrigel (MG) or in PEG-RGD (PEG) and maintained in expansion medium (EM) or differentiation medium (DM). **(d)** Liver organoids grown in physiologically-stiff hydrogels were transplanted in soft hydrogels immediately before inducing the differentiation. **(e)** Liver organoids maintained in DM in PEG-RGD hydrogels. E-cadherin was used to visualize cell borders. The square indicates a bi-nucleated cell. Scale bar 10 μm. **(f)** Representative confocal immunofluorescence images of Albumin (ALB) or Glutamine synthase (GLUL) and Phalloidin (ACTIN). Organoids were derived and maintained as in Supplementary Figure 2c. Scale bar

10 μm. Graphs show individual data points derived from n=3 independent experiments and means ± SEM. n.s. (not significant). *$P<0.05$, unpaired Student's two-tailed t-test. Source data are provided as a Source Data file.

Supplementary Figure 3:

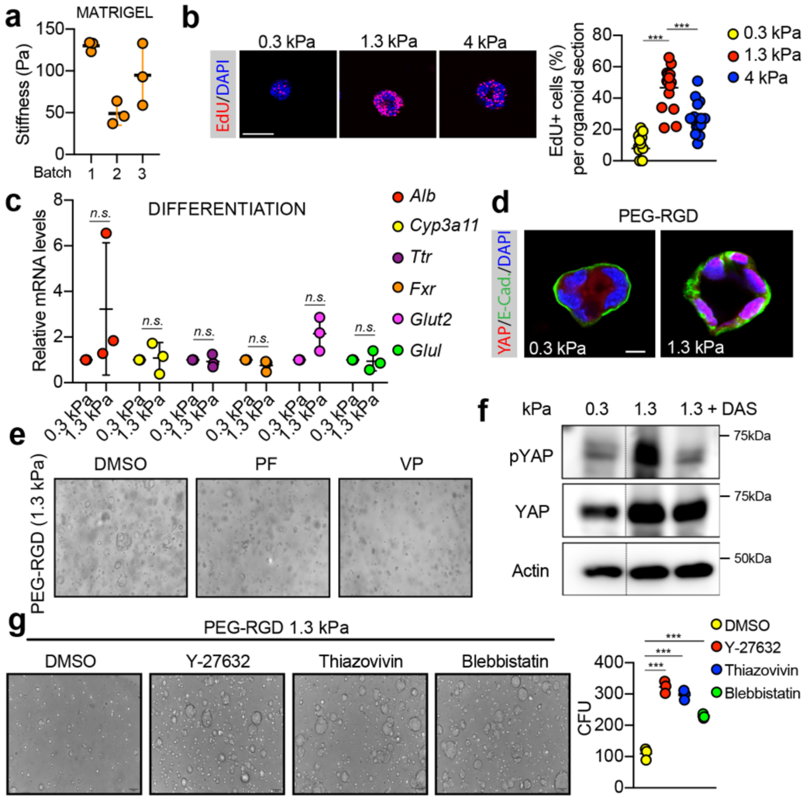

Supplementary Figure 3. Effect of PEG stiffness on liver organoids | (a) Stiffness measurements of three different Matrigel batches. **(b)** Representative images (left) and quantification (right) of EdU staining of liver organoids, 3 days after embedding in PEG-RGD hydrogels with indicated stiffness. Graphs show individual data points and means ± SEM (n=15 organoids), unpaired Student's two-tailed t-test. ***P<0.001 (P<0.0001; <0.0001). Scale bar: 100 µm. **(c)** Gene expression was analysed by qRT-PCR in liver organoids embedded in soft (0.3 kPa) or physiologically-stiff (1.3 kPa) PEG-RGD hydrogels and maintained in DM. Graphs show individual data points and means ± SEM (n=3 biologically independent experiments). Unpaired Student's two-tailed t-test. n.s. (not significant). **(d)** Representative images of YAP subcellular localization in liver organoids 1 day after embedding in soft (0.3 kPa) or physiologically-stiff (1.3 kPa) PEG-RGD hydrogels. **(e)** Representative images relative to Fig. 3e. **(f)** Western blot showing YAP phosphorylation in mouse liver progenitor cells 6

hours after embedding in soft (0.3 kPa) and physiologically-stiff (1.3 kPa) PEG-RGD hydrogels. **(g)** Matrigel-derived liver progenitor cells were embedded in physiologically-stiff PEG-RGD hydrogels and cultured in expansion medium containing ROCK inhibitors (Y-27632 and Thiazovivin) or Blebbistatin. Colony forming units (CFU) were quantified after 3 days. Graphs show individual data points and means ± SEM (n=3 biologically independent experiments), one way Anova. ***P<0.001 (P<0.0001; P<0.0001; P<0.0001). Micrographs are representative of three independent experiments. Western blots were performed pooling organoids derived from 3 different mice and repeated two times. Source data are provided as a Source Data file.

Supplementary Figure 4:

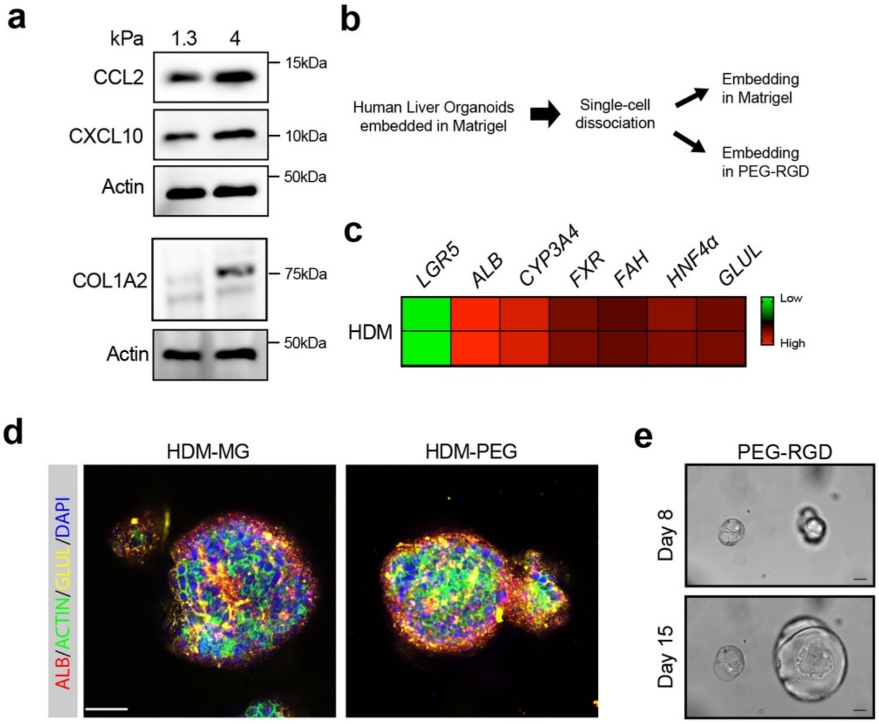

Supplementary Figure 4. Human liver organoids in PEG hydrogels | (a) Western blot of mouse liver progenitor cells 6 days after embedding in PEG-RGD hydrogels with indicated stiffness. **(b)** Schematic representation of the protocol. **(c)** Gene expression was analysed by qRT-PCR in human liver organoids embedded in PEG-RGD hydrogels and maintained in human differentiation medium. The heatmap represents ΔCt values as described in the method section and shows results from two biological replicates. **(d)** Representative confocal immunofluorescence image of Albumin and GLUL in human liver organoids cultured in human differentiation medium (HDM). **(e)** Human liver organoids were frozen-thawed and monitored in time. Days post-thawing are indicated. Scale bar: 100 μm. Micrographs are representative of three independent experiments. Western blots were performed pooling organoids derived from 3 different mice and repeated two times. Source data are provided as a Source Data file.

Supplementary Table 1. Primers used for qRT-PCR analysis.

Gene	Species	Forward	Reverse
Cyr61	Mouse	CTGCGCTAAACAACTCAACGA	GCAGATCCCTTTCAGAGCGG
Ctgf	Mouse	GCTTGGCGATTTTAGGTGTC	CAGACTGGAGAAGCAGAGCC
Cyp3a11	Mouse	TGGTCAAACGCCTCTCCTTGCTG	ACTGGGCCAAAATCCCGCCG
Alb	Mouse	GCGCAGATGACAGGGCGGAA	GTGCCGTAGCATGCGGGAGG
Ttr	Mouse	ATGGTCAAAGTCCTGGATGC	AATTCATGGAACGGGGAAAT
Fxr	Mouse	ACAGCTAATGAGGACGACAG	GATTTCCTGAGGCATTCTCTG
Glut-2	Mouse	GACCGTGGTGAACCTGCTAT	TGCGGGAATCATAGTCCTTC
Glul	Mouse	CAAGTGTGTGGAAGAGTTACCTGAGT	TGGCAACAGGATGGAGGTACA
Lxr	Mouse	TGCCATCAGCATCTTCTCTG	GGCTCACCAGCTTCATTAGC
Lgr5	Mouse	ATTCGGTGCATTTAGCTTGG	CGAACACCTGCGTGAATATG
Gapdh	Mouse	GGAGAGTGTTTCCTCGTCCC	ACTGTGCCGTTGAATTTGCC
LGR5	Human	GACTTTAACTGGAGCACAGA	AGCTTTATTAGGGATGGCAA
ALB	Human	CTGCCTGCCTGTTGCCAAAGC	GGCAAGGTCCGCCCTGTCATC
CYP3A4	Human	TGTGCCTGAGAACACCAGAG	GTGGTGGAAATAGTCCCGTG
FXR	Human	ACAGCTGCGACAGATTGGTT	TCAGAGGGGTTAGACAGCTCA
HNF4A	Human	CGTGCTGCTCCTAGGCAATGAC	ACGGACCTCCCAGCAGCATCT
GLUL	Human	GCTGGTGTAGCCAATCGTAGC	GGCTTCTGTCACCGAAAAGG
GAPDH	Human	TACTAGCGGTTTTACGGGCG	TCGAACAGGAGGAGCAGAGAG
Tnfa	Mouse	GTAGCCCACGTCGTAGCAAAC	AGTTGGTTGTCTTTGAGATCCATG
Mmp3	Mouse	GGAAATCAGTTCTGGGCTATACGA	TAGAAATGGCAGCATCGATCTTC
Ccl2	Mouse	AGGTCCCTGTCATGCTTCTG	GCTGCTGGTGATCCTCTTGT
Col1a2	Mouse	AAGGAGTTTCATCTGGCCCT	AGCAGGTCCTTGGAAACCTT
Mmp2	Mouse	AACTACGATGATGACCGGAAGTG	TGGCATGGCCGAACTCA
Tgfb1	Mouse	GAATGACGGTGCGCAACTCT	CAGCCCCAATAACCGTATGAA
Cxcl1	Mouse	TCTCCGTTACTTGGGGACAC	CCACACTCAAGAATGGTCGC
Mmp13	Mouse	ACAAAGATTATCCCCGCCTCATA	CACAATGCGATTACTCCAGATACTG

Cxcl10	Mouse	CCACGTGTTGAGATCATTGCC	GAGGCTCTCTGCTGTCCATC
Cygb	Mouse	GCTGTATGCCAACTGCGAG	CCTCCATGTGTCTAAACTGGC
Gfap	Mouse	TCGAGATCGCCACCTACAG	GTCTGTACAGGAATGGTGATGC
Acta2	Mouse	GTCCCAGACATCAGGGAGTAA	TCGGATACTTCAGCGTCAGGA
Lrat	Mouse	TACACAGGCCTGGCATCATA	TCCACAAGCAGAATGGGATA
Clec4f	Mouse	ACTGAAGTACCAAATGGACAATGTT AGT	GTCAGCATTCACATCCTCCAGA
Vsig4	Mouse	TCACCTATGGCCACCCCACC	AGGCGGCCTCTGTACTTTGCCT
Stab2	Mouse	TGTCCAGACGGCTACATCAA	CCAGGGATATCCAGGACGTA
Lyve1	Mouse	CCTCCAGCCAAAAGTTCAAA	TCCAACACGGGGTAAAATGT

Supplementary Table 2. Patient clinical information.

Sex	Age	Diagnosis	Cirrhosis
Male	58	ASH, alcoholic steatohepatitis	YES
Female	36	AFLD, alcoholic fatty liver disease	NO
Male	43	NAFLD, nonalcoholic fatty liver disease	NO
Female	45	NASH, nonalcoholic steatohepatitis	NO
Male	35	NASH, nonalcoholic steatohepatitis	NO
Female	73	Normal	NO
Male	64	ASH/NASH	NO
Male	37	DILI, drug induced liver injury	NO
