# Supplementary material for: Mechano-modulatory synthetic niches for liver organoid derivation
Source: Nat Commun. 2020 Jul 10;11:3416. doi: 10.1038/s41467-020-17161-0 (PMC7351772; doi:10.1038/s41467-020-17161-0)
Supplement: Supplementary file 3 — Reporting Summary [file 41467_2020_17161_MOESM3_ESM.pdf]

nature research

Corresponding author(s): Matthias P. Lutolf and Kristina Schoonjans

Last updated by author(s): May 27, 2020

Reporting Summary

Nature Research wishes to improve the reproducibility of the work that we publish. This form provides structure for consistency and transparency in reporting. For further information on Nature Research policies, see Authors & Referees and the Editorial Policy Checklist.

Statistics

For all statistical analyses, confirm that the following items are present in the figure legend, table legend, main text, or Methods section.

n/a	Confirmed	
☐	☒	The exact sample size (*n*) for each experimental group/condition, given as a discrete number and unit of measurement
☐	☒	A statement on whether measurements were taken from distinct samples or whether the same sample was measured repeatedly
☐	☒	The statistical test(s) used AND whether they are one- or two-sided<br>*Only common tests should be described solely by name; describe more complex techniques in the Methods section.*
☐	☒	A description of all covariates tested
☐	☒	A description of any assumptions or corrections, such as tests of normality and adjustment for multiple comparisons
☐	☒	A full description of the statistical parameters including central tendency (e.g. means) or other basic estimates (e.g. regression coefficient) AND variation (e.g. standard deviation) or associated estimates of uncertainty (e.g. confidence intervals)
☐	☒	For null hypothesis testing, the test statistic (e.g. *F*, *t*, *r*) with confidence intervals, effect sizes, degrees of freedom and *P* value noted<br>*Give P values as exact values whenever suitable.*
☒	☐	For Bayesian analysis, information on the choice of priors and Markov chain Monte Carlo settings
☒	☐	For hierarchical and complex designs, identification of the appropriate level for tests and full reporting of outcomes
☒	☐	Estimates of effect sizes (e.g. Cohen's *d*, Pearson's *r*), indicating how they were calculated

Our web collection on statistics for biologists contains articles on many of the points above.

Software and code

Policy information about availability of computer code

Data collection	No customized software were used; Light cycler 480 (Roche) real-time PCR system and associated software were used to collect real-time PCR data; microscope data were collected using Zeiss ZEN and associated software. Image J (1.50b) was used for image processing and colony quantification.
Data analysis	No custom-made software was used for data analysis. Statistical analyses were performed using Graphpad Prism 7.

For manuscripts utilizing custom algorithms or software that are central to the research but not yet described in published literature, software must be made available to editors/reviewers. We strongly encourage code deposition in a community repository (e.g. GitHub). See the Nature Research guidelines for submitting code & software for further information.

Data

Policy information about availability of data

All manuscripts must include a data availability statement. This statement should provide the following information, where applicable:
- Accession codes, unique identifiers, or web links for publicly available datasets
- A list of figures that have associated raw data
- A description of any restrictions on data availability

All summary or representative data generated and supporting the findings of this study are available within the paper. Source data are provided as a Source Data file.

Field-specific reporting

Please select the one below that is the best fit for your research. If you are not sure, read the appropriate sections before making your selection.

☒ Life sciences ☐ Behavioural & social sciences ☐ Ecological, evolutionary & environmental sciences

For a reference copy of the document with all sections, see nature.com/documents/nr-reporting-summary-flat.pdf

Life sciences study design

All studies must disclose on these points even when the disclosure is negative.

Sample size	No statistical method was used to predetermine the sample size. Sample sizes in this study were estimated based on our experience and previous publications (i.e. Gjorevski et al. Nature 2016).
Data exclusions	No data were excluded for all figures.
Replication	All experiments were reliably reproduced and results are represented as mean and SD or SEM, as indicated in figure legends. Student's t test or Anova were utilized to compare two or more independent conditions. A P value of 0.05 or less was considered statistically significant, which is described in the methods section. The experiments were repeated at least 3 times or with at least 3 different donors to control biological variations.
Randomization	Randomization of animals is not relevant to this study.
Blinding	Investigators were not blinded to the studies. Organoid formation quantification was performed by at least 2 investigators.

Reporting for specific materials, systems and methods

We require information from authors about some types of materials, experimental systems and methods used in many studies. Here, indicate whether each material, system or method listed is relevant to your study. If you are not sure if a list item applies to your research, read the appropriate section before selecting a response.

Materials & experimental systems

n/a	Involved in the study	
☐	☒	Antibodies
☒	☐	Eukaryotic cell lines
☒	☐	Palaeontology
☐	☒	Animals and other organisms
☐	☒	Human research participants
☒	☐	Clinical data

Methods

n/a	Involved in the study	
☒	☐	ChIP-seq
☒	☐	Flow cytometry
☒	☐	MRI-based neuroimaging

Antibodies

Antibodies used	Primary antibodies used: Epcam (1:50, eBioscience, G8.8), Krt19 (1:100, ab15463), E-cadherin (1:100, Cell Signaling, 24E10), Sox9 (1:50, Millipore, AB5535), Albumin (1:50, R&D systems, MAB1455), Hnf4α (Santa Cruz, C19), ZO-1 (Invitrogen, 61-7300); YAP (Cell Signaling, 4912S); CXCL10 (RD system AF-466-NA, 1:1000), CCL2 (Novus Biologicals NBP2-22115, 1:1000), COL1A2 (Santa Cruz, 1:1000), YAP1 (Santa Cruz sc101199, 1:500), phospho-YAP1Y357 (Abcam ab62751, 1:1000), and ACTIN (Santa Cruz sc47778, 1:5000). Secondary antibodies: Alexa 488 donkey-α-rabbit, Alexa 568 donkey-α-mouse, Alexa 647 donkey-α-goat (1:1000 in blocking solution; Invitrogen).
Validation	Antibody validation was performed by the manufacturer. Extensive antibody-validation data for each species and application, with relevant citations, are provided in each vendor's product website.

Animals and other organisms

Policy information about studies involving animals; ARRIVE guidelines recommended for reporting animal research

Laboratory animals	Liver tissues were harvested from 8-12 weeks old euthanized C57BL/6J male mice.
Wild animals	No wild animals were used in this study.
Field-collected samples	No field-collected samples were used.

Ethics oversight	All the animal experiments were authorized by the Veterinary Office of the Canton of Vaud, Switzerland under the license authorization no. 3263.

Note that full information on the approval of the study protocol must also be provided in the manuscript.

Human research participants

Policy information about studies involving human research participants

Population characteristics	Information on human participants are listed in Supplementary Table 2.
Recruitment	Human tissues were obtained from patients undergoing diagnostic liver biopsy at the University Hospital Basel. No biases that can impact on results have been applied.
Ethics oversight	Written informed consent was obtained from all patients. The study was approved by the ethics committee of the northwestern part of Switzerland (Protocol Number EKNZ 2014-099).

Note that full information on the approval of the study protocol must also be provided in the manuscript.
